# Home-based management of knee osteoarthritis during COVID-19 pandemic: literature review and evidence-based recommendations

**DOI:** 10.1186/s40634-020-00271-5

**Published:** 2020-07-19

**Authors:** Theofilos Karasavvidis, Michael T. Hirschmann, Nanne P. Kort, Ioannis Terzidis, Trifon Totlis

**Affiliations:** 1 School of Medicine, Faculty of Health Sciences, Aristotle University of Thessaloniki, University Campus, 54124 Thessaloniki, Greece; 2 Department of Orthopaedic Surgery and Traumatology, Kantonsspital Baselland (Bruderholz, Liestal, Laufen), 4101 Bruderholz, Basel, Switzerland; 3 CortoClinics, Steeg 6E, 5482 WN Schijndel, The Netherlands; 4 Thessaloniki Minimally Invasive Surgery Orthopaedic Center, St. Luke’s Hospital, 55236 Panorama, Greece

**Keywords:** Osteoarthritis, Knee, COVID-19, Home-based, Exercise, Diet, Physical therapy, Corrective devices, Total knee arthroplasty

## Abstract

**Purpose:**

To provide evidence-based recommendations for patients with severe knee osteoarthritis (OA), who had their knee surgery postponed due to the COVID-19 pandemic.

**Methods:**

PubMed/Medline, Scopus and Cochrane Central databases were systematically reviewed for studies reporting outcomes of home-based treatments for knee OA. Due to between-study differences in treatment strategy and reporting methods the results were not pooled and findings of the current review were presented in a narrative manner.

**Results:**

The comprehensive literature search yielded 33 eligible studies that were included in this review. Management is performed at home and consists of exercise, proper nutrition, physical therapy and use of corrective and assistive orthotics. Virtual education on self-management strategies should be part of coping with knee OA. Initiating an exercise programme involving gymnastics, stretching, home cycling and muscle strengthening is highly recommended. Obese patients are encouraged to set weight loss goals and adopt a healthy diet. Potential benefits but weak evidence has been shown for the use of knee braces, sleeves, foot orthotics or cushioned footwear. Walking aids may be prescribed, when considered necessary, along with the provision of instructions for their use.

**Conclusion:**

When bridging the time to rescheduled surgery, it is essential to use appropriate home-based tools for the management of knee OA if pain is to be reduced and need for analgesics or opioid use is to be diminished while maintaining or even improving the functioning and avoiding further limitation of range of motion and subsequent muscular atrophies. Finally, none of these treatments may completely substitute for the life-changing effect of a total knee arthroplasty in patients with severe knee OA. Hence, the subsequent goal is to gradually and safely reinstate elective surgery.

## Introduction

Osteoarthritis (OA) is considered the most common type of arthritis and the most prevalent joint disease in adults [1]. Total knee arthroplasty (TKA) has been proven to be an exceptional and dependable treatment for end stage knee OA, with satisfactory clinical outcomes up to 20-year follow-up [2]. The beneficial influence of knee arthroplasty on symptom management, social life, patient satisfaction and overall health promotion is indisputable [3].

Coronavirus disease 2019 (COVID-19) has evolved as a pandemic throughout the world; it constitutes an unprecedented challenge for the personal and professional lives of healthcare providers and has severe socio-economic consequences for most countries [4]. During these efforts to delay the spread of the virus and protect patients and staff, hospitals have had to stop most non-COVID-related activity and postpone the majority of elective surgeries, including knee arthroplasties [5–7]. Notably, the COVID-19 survey of European Knee Associates (EKA) and European Hip Society (EHS) members in April 2020 demonstrated that 92.6% of surgeons had stopped total joint arthroplasties, while the American Association of Hip and Knee Surgeons (AAHKS) COVID-19 survey showed similar data in the USA, where 92% of hospitals had stopped elective surgeries [8, 9]. Likewise, Asian orthopedic surgeons had postponed all surgeries requiring > 23 h of hospitalization [10]. In addition, knee surgery could increase the risk of complications for ageing patients [4] given that they are at increased risk of severe COVID-19. However, delaying elective TKA in patients with severe OA may lead to increasing joint pain, less mobility, functional limitations and increased use of analgesic and opioids. More recently, most countries have begun to consider a restart of elective surgeries, including knee arthroplasties [11, 12]. However, the safety of patients and medical staff remains crucial and as such, the gradual reinstatement of elective joint replacements will be an ongoing and time-consuming challenge [13].

The aim of the present review is to provide a guideline for physicians to lead the patients with knee OA, who had their knee surgery postponed due to COVID-19, through their home-based treatment. Even though current sanitary measures such as social distancing seem to contribute to a flattening of the curve, they constitute burdens for patients suffering from severe knee OA, who often cannot come into direct personal contact with their physician or physical therapist responsible for medication prescription or physical therapy sessions respectively. It is against this background that this review focuses on available home-based non-pharmacological options with the goal of mitigating pain, improving function and preventing further progression of the disease.

## Methods

### Study selection and data extraction

Systematic searches were conducted in PubMed/Medline, Scopus and Cochrane Central according to the Preferred Reporting Items for Systematic Reviews and Meta-Analyses (PRISMA) guidelines [14]. The databases were reviewed using the following search terms in several combinations: “home-based”, “exercise”, “physical activity”, “training”, “strengthening”, “aerobic”, “blood flow restriction”, “BFR”, “diet”, “weight loss”, “nutrition”, “physical therapy”, “brace”, “orthotics”, “footwear”, “walking aids”, “cane”, “walker”, “osteoarthritis” and “knee”. All publications up to 26 May 2020 were searched. Two authors, independently and blind to each other, screened the titles and abstracts of all the articles identified. If the abstract was unavailable, the paper was excluded. In the event of disagreement, a consensus was reached by discussion, if needed with the intervention of the senior author. Randomized controlled trials (RCTs), prospective or retrospective analyses as well as systematic reviews and meta-analyses, reporting on conservative management of knee OA, were included in this review. A study was excluded from this review, if the treatment described was not possible to be performed at home.

Two investigators independently extracted the relevant data from eligible studies. All disagreements were resolved following discussion and the final decision was reached by consensus with the addition of a third reviewer. Where required, the corresponding authors were contacted for additional information. Data were extracted from each article included and entered into a Microsoft Excel spreadsheet for analysis. Pertinent information extracted included author, date and journal of publication, study design (and level of evidence), and patient demographics (mean age, mean follow-up, total and group’s number of patients, outcome). However, due to heterogeneity of available data in treatment strategy and reporting methods, it was decided to present the review in a narrative manner. Primary studies in the current literature reporting on conservative management of knee OA are characterized by a lot of different treatment protocols and outcomes measures. For this reason, it was not possible to synthesize and systematically analyze the existing data by pooling the results of each study, so as to produce the overall result of a treatment option.

## Results

### Literature search

**Fig. 1:**
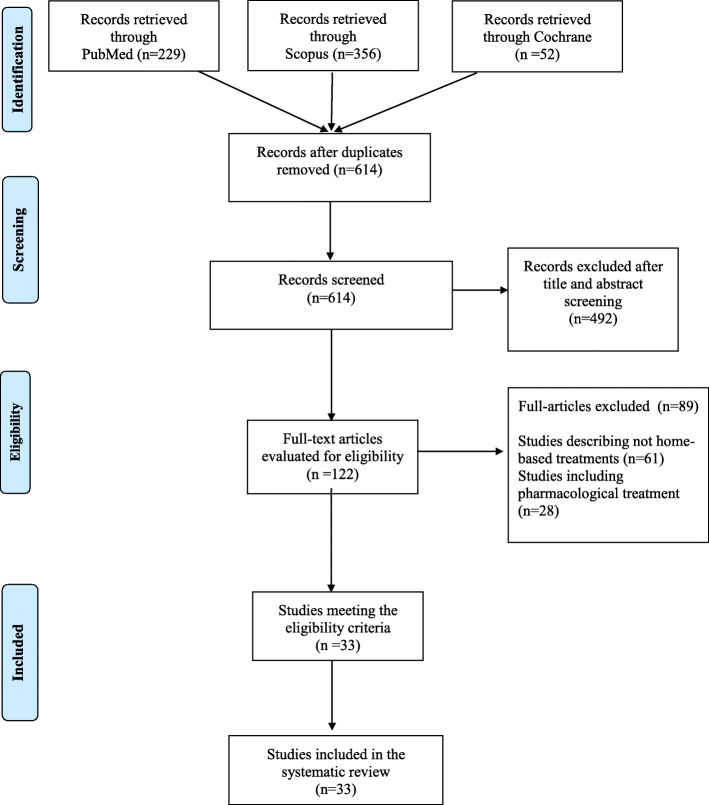
PRISMA flow chart

The literature search yielded 614 potentially relevant records, after duplicates were removed. After screening titles and abstracts, 122 articles were retrieved for full-text evaluation. Eighty-nine studies were excluded for the following reasons: a) studies describing treatments not possible to be performed at home and b) studies including pharmacological treatment. Thirty-three studies met the predetermined eligibility criteria and were included in this review. The PRISMA flowchart was applied to illustrate the step-by-step selection process (Fig. 1).

#### Exercise and physical activity

Clinical guidelines suggest that exercise is the primary non-pharmacologic treatment of knee OA [15, 16]. The most common types of exercise used for treating knee OA are low impact aerobic exercise like walking or preferably cycling and resistance training in combination with proprioceptive and range of motion activities [17, 18]. There is sufficient evidence for the favorable effect of exercise on pain reduction and physical function improvement [19, 20]. As such, patients with knee OA should be encouraged to undertake aerobic, muscle strengthening and range of motion exercises in their daily schedule, while limiting the movements that cause extra pain and axial joint loading [21]. Exercise benefits patients by inducing muscular hypertrophy, strengthening and increasing blood flow and joint lubrication [22].

Three meta-analyses including 60, 48 and 20 randomized controlled trials respectively, demonstrated a beneficial effect of aerobic, resistance and performance exercise on knee OA, but did not recommend the most favorable exercise regimen [23–25]. Very few studies have compared different exercise intensity, duration and progression and, due to different protocols used, the optimal combination still remains uncertain with no type of exercise shown to be better than another [19, 26, 27]. Mixed programmes are recommended when it comes to the type of exercise [15].

Quadriceps strengthening exercises, strength training of the hip or lower limb and aerobic training have favorable effects regarding pain and knee function [19, 28]. Range of motion exercise prevents development of contractures while periarticular muscle strengthening improves symptoms [20, 27]. Stabilization exercises may be used, since OA often leads to instability in the knee due to biomechanical imbalances [29, 30]. The aforementioned exercises could incorporate progression over time, which has been shown to reduce pain more effectively, when compared with non-progressive programmes [27, 31]. Running on hard surfaces, jumping, stair climbing and squatting should be limited [32]. The exercise regimen may be adapted according to the pain experienced and functional limitations.

Blood flow restriction (BFR) training is commonly used in athletes to improve hypertrophy in biceps brachii and quadriceps femoris muscles [33]. BFR training uses low resistance exercise and can be effective in improving function and muscle hypertrophy, when conventional quadriceps strengthening programmes with high resistance exercise exacerbate knee symptoms [34]. This concept may contribute to the home-based management of knee OA by ameliorating the quadriceps weakness that often comes with this degenerative condition without provoking training-related knee pain [34]. BFR has been found to be a safe alternative for older adults with knee OA to reduce pain and improve function [35]. A randomized controlled trial from 2018 demonstrated that BFR is able to improve pain, while inducing less knee joint stress in patients with OA compared to resistance training alone [36]. The literature suggests that adherence to physical activity is often directly dependent on the use of exercise plans or log books containing feedback on progress and the effect on pain [37, 38].

Most importantly, the clinical status of patients with knee OA during the COVID-19 pandemic, can be improved if patients are contacted regularly by phone or participate in live virtual sessions with their physician or physical therapist [16, 17, 39]. Every exercise programme should be supervised during the first days of implementation at least.

#### Diet and weight loss

During the pandemic patients with knee OA mostly stay at home; they should therefore pay special attention to their weight. Weight gain, together with decreased strength of surrounding musculature, may increase the load on the knee [40]. There is evidence that for each kilogram (kg) of weight loss, the knee experiences a 4 kg reduction in load per step and a 4800 kg reduction in compressive load for each kilometer walked [16, 41]. Individualized diet interventions with weight loss goals and involving regular follow up to assess the progress have been associated with pain and functional improvements in overweight patients with severe knee OA [41, 42]. European League Against Rheumatism (EULAR) recommendations on how to control your weight focus on calorie intake, intake of fats and sugar and portion size in combination with self-monitoring and the setting of weight-loss goals [15].

A meta-analysis assessed the changes regarding pain and function in obese patients with knee OA who achieved a weight loss. It demonstrated a significant reduction in disability when weight was reduced > 5.1% over a 20-week period or at the rate of > 0.24% reduction per week [43]. Another study evaluated patients with knee OA and concluded that weight gain ≥10% of body weight was associated with a worse Western Ontario and McMaster Universities Osteoarthritis Index (WOMAC) physical function score [44]. Teichtahl et al. showed that obese patients with OA who lost as little as 1% of their body weight were able to reduce the amount of medial femorotibial cartilage volume loss [45].

A 18 month RCT demonstrated that the combination of weight loss and exercise is more effective in managing knee OA than either one alone, with patients in the combined diet and exercise group achieving more weight loss and greater reductions in WOMAC pain scores and IL-6 levels [41]. Education on OA management along with information on physical exercises and weight loss by virtual visits to the physician could reinforce the effectiveness of exercise and weight loss programmes when implemented simultaneously [46, 47].

#### Physical therapy

A symptomatic patient suffering from severe end-stage knee OA may benefit from referral to a physical therapist for video instructions and supervision [21]. Initial supervised sessions have been shown to act favorably on patient adherence to home exercise programmes, resulting in less pain and improved functioning [48, 49].

There is little evidence to support the use of thermal packs or ice packs. However, due to the fact that they are accessible and affordable for most patients, thermal modalities are included in OA treatment guidelines for managing pain [16]. Heat may be applied through warm water or heat packs and could be used at the same time as stretching during painful episodes [16]. Cryotherapy is typically implemented with ice packs for managing acute episodes of inflammation and pain, without any significant effects demonstrated on pain coming from knee OA [21, 50]. Alternation between heat and cold therapies (e.g. starting with a hot pack and ending with a cold one) is often suggested by physical therapists in daily practice; however there is insufficient data in the literature to support potential benefits of this technique [50].

#### Braces, orthotics and footwear

Corrective devices, such as knee braces, orthotics and footwear may be effective for management of knee OA symptomatology [40]. Knee bracing and foot orthoses may contribute to countering the pain coming from excessive knee adduction moment during walking that has been associated with severe radiographic knee OA [51]. Both of these devices constitute effective means in the home-based armamentarium to tackle pain and joint stiffness [52].

Knee braces are designed to alter contact pressures, especially with uni-compartmental knee OA, by exerting either valgus or varus force [53]. A knee brace can improve stability, reduce the muscular contraction needed to stabilize the affected knee and minimize the risk of falling [21, 54]. Brouwer et al. also demonstrated improved pain scores and walking tolerance at 1 year with knee off-loader braces, especially in the medial compartment OA group [55]. It is suspected that a brace acts by improving the biomechanical axis of the deformity or the perception of instability [32]. Hussain et al. suggested that a patient with uni-compartmental OA is the ideal one to benefit from an orthosis in terms of pain and function [32]. More high-quality studies are warranted to elucidate which subset of knee OA patients are likely to benefit from knee braces, sleeves and orthotics.

Appropriate footwear is recommended in every patient with knee OA. Shoes could affect by acting as shock absorbers or controlling foot pronation [15, 52]. Turpin et al. demonstrated that the use of shoes with shock-absorbing insoles for 1 month reduced pain and improved functioning [56]. Shoes requirements include shock-absorbing soles, support for the arches, no raised heel and a size big enough to give space for the toes [52]. In patients with medial knee OA, lateral wedge insoles may reduce pain, improve ambulation by reducing knee adduction moment and therefore decrease joint stress; however studies have shown controversial results not always ending in significant benefits [15, 54, 57].

#### Walking aids

Walking aids and assistive technology at home should be considered as a safe alternative in patients with knee OA, since the value of some of these measures has an immediate impact in individual patients [15]. Reduction of pain can be achieved with use of walking aids and patients should be taught the optimal use of a cane or crutch in the contralateral hand, while wheeled walkers are ideal for those suffering from bilateral pathologies [16, 21].

Canes are considered affordable means to unload the affected joint, provide stability and potentially reduce pain. Walkers are suggested for patients who require maximum assistance, particularly for the elderly. Patients must have good arm strength bilaterally. However, patients may become dependent on walkers; thus they should typically be prescribed for the pre-operative or early post-operative period or in severe disease circumstances [21]. Finally, it is specified in the literature that walking aids at home play a crucial role in management of knee OA, since 90% of adult people suffering from severe knee pain report the use of canes [58–60].

## Limitations

The results of the present review should be interpreted in the context of several limitations. First, due to between-study differences in treatment strategy and reporting methods, results could not be pooled. Subsequently, risk of bias of the included studies as well as publication bias of our outcomes could not be assessed. Additionally, the circumstances established by the pandemic led us to find conservative alternatives for the management of knee OA at all stages, and this increases the risk of selection bias for the current review. However, by following the PRISMA guidelines during our search strategy and data extraction, we attempted to provide high-quality evidence-based recommendations for the patients, who have had their knee surgery postponed during COVID-19 pandemic.

## Conclusion

According to this review, under current circumstances established by the pandemic, virtual education on self-management strategies could be part of tackling knee OA. Initiating an exercise programme involving aerobic activity (preferably cycling), strengthening and flexibility is recommended. Obese patients are encouraged to set weight loss goals and adopt healthy nutrition habits. The literature indicates that potential benefits may be gained from the use of knee braces, sleeves, foot orthoses and appropriate footwear but the evidence is weak. Walking aids may be prescribed, when considered necessary, along with the instructions of use. Further research is required to clarify which is the most efficient non-pharmacological, home-based treatment plan for the management of knee OA. Finally, it is crucial to take into consideration that none of these treatments may completely substitute for the life-changing effect of a TKA in patients with severe knee OA. Of course, the ultimate goal is to gradually and safely reinstate elective surgeries with the minimum impact on patients that had their surgery postponed.

## Data Availability

Data sharing is not applicable to this article as no datasets were analysed during the current study.

## Abbreviations

AAHKS: American Association of Hip and Knee Surgeons
BFR: Blood flow restriction
COVID-19: Coronavirus disease 2019
EHS: European Hip Society
EKA: European Knee Associates
EULAR: European League Against Rheumatism
kg: Kilogram
OECD: Organization for Economic Co-operation and Development
OA: Osteoarthritis
RCTs: Randomized controlled trials
SARS-CoV-2: Severe acute respiratory syndrome coronavirus 2
TKA: Total knee arthroplasty
WOMAC: Western Ontario and McMaster Universities Osteoarthritis Index
